# Validation of the behavioral regulation in cycling to and from school scale among students from Germany

**DOI:** 10.3389/fspor.2026.1701435

**Published:** 2026-02-11

**Authors:** Dorothea M. I. Schönbach, Adilson Marques, Miguel Peralta, Dorota Kleszczewska, Anna Dzielska, Rafael Burgueño, Francisco Javier Huertas-Delgado, Yolanda Demetriou

**Affiliations:** 1 Department of Rehabilitation and Sports Medicine, Hannover Medical School, Hanover, Germany; 2 TUM School of Medicine and Health, Technical University of Munich, Munich, Germany; 3 Centro Interdisciplinar de Performance Humana (CIPER), Faculdade de Motricidade Humana, Universidade de Lisboa, Lisbon, Portugal; 4 Núcleo de Investigación en Ciencias del Movimiento, Universidad Arturo Prat, Iquique, Chile; 5 Department of Health Sociology, Education and Medical Communication, Institute of Mother and Child, Warsaw, Poland; 6 Department of Child and Adolescent Health, Institute of Mother and Child, Warsaw, Poland; 7 Department of Didactics of Languages, Arts and Sport, University of Malaga, Malaga, Spain; 8 Teacher Training Centre La Inmaculada, University of Granada, Granada, Spain; 9 Institute of Sports Science, University of Tübingen, Tübingen, Germany

**Keywords:** autonomous motivation, bicycling to school, measurement instrument, organismic integration theory, self-determination theory

## Abstract

The prevalence of total physical activity among children and adolescents in Germany is reported to be low (D-) according to the country-specific Report Card from 2022. One potential strategy to increase physical activity is the promotion of cycling to and from school. To foster a more self-determined form of motivation among students, we developed a promising intervention grounded in the self-determination theory, as demonstrated in previous research. Here, we aimed to validate the “German behavioral regulation in cycling to and from school scale” focusing on construct and criterion validity as well as reliability to create a robust measurement instrument. Conducted in Southern Germany in 2021, this study involved 112 girls, 124 boys, and three students identifying as diverse (13.5 ± 1.1 years) who attended a (sub)urban secondary school that provided an intermediate and/or high educational level. We were unable to validate the original first-order six-factor model. Instead, we successfully validated a newly developed first-order three-factor model, which demonstrated a good global model fit (GFI: 0.997, SRMR: 0.0313), measurement model invariance across gender (GFI: 0.986–0.994, SRMR: 0.0426–0.0596), and internal consistency (*α*: 0.846–0.927). For this new scale, both convergent (AVE: 0.582–0.762) and discriminant validity $\left(R\;<\;\sqrt{\mathrm{AVE}}\right)\;$ were established. Concurrent criterion validity was found acceptable by the correlation between the mean value for each of the three newly developed factors and the frequency of cycling to school (*R* = 0.616) as well as the increased likelihood of cycling to school based on mean levels of autonomous (*p* < 0.001; OR = 2.5 [CI 95 for OR: 1.9, 3.3]) and controlled (*p* < 0.001; OR = 1.7 [CI 95 for OR: 1.3, 2.4]) motivation. Consequently, we concluded that our study provides a valid and reliable scale measuring behavioral regulation in cycling to and from school, by applying a first-order three-factor model. Furthermore, researchers and practitioners, such as (physical education) teachers, could use this scale to assess the motivation for cycling to and from school in order to implement and evaluate contextual and tailored interventions.

## 1 Introduction

In 2018, the United Nations reported that around half of the world's populations lived in urban areas and projected an increase to over two-thirds by 2050 (1). Goal 11 of the United Nations' 2030 Agenda for Sustainable Development calls for cities to be made sustainable, safe, resilient, and inclusive (2). Particularly in cities, sustainable means of transportation can improve air quality by reducing traffic-related air pollution and greenhouse gas emissions (3, 4) and minimize noise (4). Therewith, sustainable means of transportation helps improve the health of urban inhabitants (goal 3) (5) and counteract global warming, driven by climate change (goal 13) (6). Data suggest that if the rate of cycling in selected European cities matched the one of Copenhagen (Denmark), important economic benefits could be anticipated in terms of job creation as well as public health benefits related to the prevention of premature mortality (7). For example, cycling has positive impacts on the risk of cardiovascular disease and type 2 diabetes as well as problems related to mental health and wellbeing (8). Furthermore, cycling is affordable (9, 10) (as only a bike and helmet are required), efficient (as it is less impacted by congestion) (10), and convenient (as one is not reliant on public transport schedules) (9, 10). Cycling also provides access to locations that may be difficult to reach, such as places without public transport (9, 10) or too far to walk (10) or areas lacking parking (10). In addition, cycling can be easily integrated into daily routines such as to and from school, university or work, visiting friends and family, or shopping and leisure time destinations (9, 10).

Children and adolescents who cycle to and from school tend to be more physically active and are more likely to develop a lifelong habit (11), which could contribute to improving the poor average rating of D for overall physical activity, reflecting the situation in 57 countries as published in the Global Matrix 4.0 on physical activity for children and adolescents (12). According to this report, active travel—encompassing walking and cycling—was assigned a moderate average rating of C-. In Germany's 2022 Report Card on physical activity for children and adolescents, a poor rating was also assigned to overall physical activity (i.e., D-) and a moderate rating to active travel (i.e., C) (13).

Almost all children and adolescents aged 7–17 own a bike in Germany (i.e., 89%–98%) (14). In a previous and this study including a total of 362 students from Germany aged between 10 and 17, all students reported to be able to cycle (15, 16). Therefore, we developed a school-based randomized controlled trial aimed at the promotion of cycling to and from secondary school, thereby increasing overall physical activity among 12- to 15-year-old students from grade seven or eight with intermediate or high educational level in (sub)urban areas in Southern Germany (17). To promote a more self-determined form of motivation among students, we created a theoretical model during the systematic development process, which incorporates two of the six mini-theories of the self-determination theory (17). For the first mini-theory, we have already published the validation of the need satisfaction in cycling to and from school scale among students from Germany (15). The present publication focuses on establishing a robust instrument to measure the second mini-theory included in our theoretical model: organismic integration theory.

This self-determination theory operationalizes motivation from both quantitative and qualitative perspectives, with a particular emphasis on the latter, in contrast to classical motivational theories that conceptualize motivation as a quantitative unitary construct (18). This means that a high quantity of motivation does not necessarily ensure the adoption of the target behavior if the underlying motivation is of low quality (19). The self-determination theory distinguishes three qualities of motivation along a self-determination continuum, depending on the level of relative autonomy or willingness present in each (19). At one end of the self-determination continuum stands intrinsic motivation, which refers to displaying the behavior for its own sake, namely, for its inherent enjoyment, interest and curiosity, as well as the search for new horizons (19). At the center of this continuum lies extrinsic motivation, which refers to displaying a behavior as a means to an end (19). Depending on the content and degree of internalization (i.e., the process by which social and cultural norms are gradually incorporated into the self), the self-determination theory specifies four types of behavioral regulation within extrinsic motivation. External regulation implies the absence of internalization, given that the behavior is adopted under pressure from others (either to obtain social and material rewards or to avoid punishment). Introjected regulation entails a partial internalization, in which the behavior is adopted under self-imposed pressure to gain self-esteem for success or to avoid negative feelings (such as shame, anxiety, and guilt for failure). Identified regulation involves an almost complete internalization, in which the behavior is adopted because the person consciously recognizes its own benefits and personal value and perceives it as worthwhile. Integrated regulation refers to a complete internalization where the behavior is adopted because it aligns harmoniously with the person's identity and lifestyle. At the opposite end of the self-determination continuum—in contrast to intrinsic and extrinsic motivation—amotivation describes the complete lack of self-determination, regulation, and intention for displaying the target behavior (19). The self-determination theory postulates that intrinsic motivation, integrated regulation, and identified regulation—considered autonomous forms of motivation—would primarily lead to adaptive affective, behavioral and cognitive outcomes (20). In contrast, introjected regulation and external regulation—as controlled forms of motivation—and amotivation are theorized to contribute mainly to maladaptive outcomes (20).

Our previously conducted systematic review revealed that the self-determination theory has been disregarded to promote cycling to and from school and to evaluate such interventions (21). An instrument measuring behavioral regulation in active travel to and from school—including cycling and walking—has been validated among children, adolescents, and young adults from Spain in 2019 (22). Based on this Spanish instrument, the validation studies from Portugal (23) and Sweden (24) were developed in 2022. However, a domain-specific instrument developed to measure behavioral regulation specifically for cycling to and from school is currently lacking. Such an instrument is needed because cycling—unlike walking—requires specific skills and greater fitness levels, necessitates specific equipment (e.g., a bike and helmet), and depends on financial resources to ensure the roadworthiness and safety of equipment or to replace it with a new one as the child grows.

The purpose of this study was to provide a scale that allows to gain a deeper understanding of the motivational processes underlying cycling to and from school (e.g., when evaluating the effectiveness of an intervention), which involved the following three steps: (a) translation of the original Spanish behavioral regulation in active commuting to and from school questionnaire into German, (b) adaptation of the questionnaire to the context of cycling to and from school, and (c) validation of the newly developed scale in terms of construct and criterion validity as well as reliability among students.

## 2 Material and methods

Overall, the methodological procedure of our study followed the International Test Commission guidelines (25). For the translation of the original Spanish questionnaire (22), we used the double-translation and reconciliation procedure as described in the guidelines. This harmonization implies that two independently created forward translations were merged into a single translation by a third independent translator (DMIS), whereby any discrepancies were resolved through discussion. An additional backward translation was not expedient as the original questionnaire was only translated into English for publication and no person in our research group could speak Spanish and German. After the translation, three self-determination theory experts working in our research group were consulted to ensure the accuracy of our scale. The experts were specialized in the fields of active travel and motivation or physical activity and motivation in physical education (including scale validation). Based on their feedback, the scale was revised. Any discrepancies between the experts were resolved through discussion to reach a consensus (i.e., via an online meeting moderated by DMIS and emails). Finally, a small-scale pilot study among three 11- to 15-year-olds (including both sexes) had been conducted prior to our main study to ensure that our target group understands the instruction and possible responses of our scale. Based on their feedback, the scale was finalized. Overall, adaptations to our scale refer to the context of cycling to and from school, wording (including meaning and gender-sensitive language), moving a frequency adverb from individual items to the general opening paragraph for the sake of consistency, correction of numbering due to a mistake in the published original version, and syntax. The original number of subscales, allocation of items, and Likert scale points were retained. Considering the challenges at the time the study was conducted, the intended format was online from the beginning. The methodological procedure of our study (i.e., translation, expert consultation, and small-scale pilot study, including adaptations) is also described in the publication on the validation of the need satisfaction in cycling to and from school scale among students from Germany (15).

### 2.1 Data collection

Public and independent secondary schools, providing low, intermediate, and high educational levels and located in rural and (sub)urban areas in Northern and Southern Germany, were randomly contacted via email. Only five public schools located in Southern Germany agreed to participate in our study, two being suburban secondary schools located in small towns and providing intermediate or high educational level, and three being urban secondary schools located in cities (with one providing both intermediate and high educational level, and two only high educational level). Schools were asked to recruit students attending grades seven, eight, and nine for our study (i.e., no other inclusion/exclusion criteria, such as gender, were applied). For this purpose, schools forwarded a letter of information and an informed consent to relevant parents who signed and returned the informed consent form to us either via email or letter if their child(ren) volunteered to participate in this study. A statement by an ethics committee is not required according to the German Research Foundation when participants are informed about the study and when a study does not involve deception, a high level of emotions/stress/traumatic experiences, exceptional risks (e.g., social or physical), or vulnerable groups (26). In addition, a statement by the ethics committee from the researcher's institution has not been made mandatory for non-medical studies according to the institutional regulation (27). Also, at the time the study was conducted, no non-medical specialist group existed in the ethics committee of the researcher's institution. A non-medical specialist group was not established until 2023 (i.e., only 2 years after this study was conducted). Because of Covid-19, we struggled with recruiting enough students for our study initially. Thus, cross-sectional data were collected in two waves, occurring between May and July 2021 and between November and December 2021. In each wave, schools forwarded a reminder to parents. Data were collected online via Unipark because of Covid-19 restrictions such as homeschooling and avoidance of personal contact. Students could access our survey by a link provided in the parental letter of information and reminder. Within one of the two waves, students completed the 15-min, anonymous survey unsupervised and on their own personal time at home. At the beginning of the survey, a short explanation of the aim, its structure, and instructions on how to fill it out were given. No compensation was provided for study participation.

### 2.2 Measurement instruments

Our survey started with a 12-item questionnaire on sociodemographic characteristics (i.e., age, gender, zip code of school to categorize urbanization levels into small town/suburban area with 5,000–20,000 inhabitants and city/urban area with more than 100,000 inhabitants, educational level, grade, bike ownership, ability to cycle, car ownership within family, usual mode of transportation to school, days per week of cycling to school usually, shortest cycling distance between home and school in kilometer(s), and minutes to cycle from home to school).

The questionnaire was followed by two adapted scales, of which one measured behavioral regulation and the other need satisfaction in cycling to and from school (15). For the assessment of behavioral regulation in cycling to and from school, students were asked to complete the beginning of a sentence “I normally cycle or would cycle to and from school because…” by rating 23 end of records on a five-point Likert scale (1 = do not agree at all, …, 5 = completely agree). The 23 end of records stand for intrinsic motivation, extrinsic motivation (further divided into integrated, identified, introjected, and external) and amotivation, reflecting the organismic integration theory of the self-determination theory. The end of records were provided in an unsystematic order. All motivational forms consist of four items each, apart from the subscale identification with three items. Following this, the mean value for each motivational form is based on the respective number of items within each subscale. In Supplementary Table A.1, our German behavioral regulation in cycling to and from school scale and its English translation can be found.

### 2.3 Analysis

As different opinions exist on how to manage ordinal data based on Likert scales, a statistician consulted before data analysis recommended acknowledging and considering the ordinal origin of our variables.

To analyze descriptive data, IBM SPSS Statistics (RRID: SCR_016479), V.29.0, was used. Mann–Whitney *U* tests were calculated for our ordinal data to examine differences between girls and boys regarding the mean value for each factor of our tested models.

We considered the order of importance of measurement properties when analyzing our behavioral regulation in cycling to and from school scale (i.e., construct validity via structural, convergent/discriminant validity and measurement invariance as well as reliability via internal consistency for internal structure, and concurrent criterion validity), which is in line with the COnsensus-based Standards for the selection of health Measurement INstruments (28).

#### 2.3.1 Construct validity

IBM SPSS Amos (RRID: SCR_022686), V.26.0, was used to test the internal structure of our behavioral regulation in cycling to and from school scale. A first-order six-factor analysis and a second-order confirmatory factor analysis on the multidimensional scale (29) were performed, which is an indicator for structural validity. The first-order six-factor model analyzes the subscales of the six behavioral regulations, and the second-order model analyzes the categorization of subscales into autonomous motivation (i.e., intrinsic, integration, and identification), controlled motivation (i.e., introjection and external), and amotivation (30). Because of issues regarding measurement properties for the first-order six-factor model (fully discussed in Section 4.2.1), we decided to conduct a reanalysis for a first-order three-factor model, including 12 items (i.e., four items each for autonomous motivation, controlled motivation, and amotivation, respectively). A similar model has also been successfully evaluated in the Spanish, Portuguese, and Swedish validation study (22–24). The 12 items were chosen based on the results from the analysis of the first-order six-factor model (i.e., removal of the subscale introjection and selection of the four best items within the subscales intrinsic, integration, and identification based on standardized regression weights obtained through confirmatory factor analysis). Following this procedure, the factor autonomous motivation included the items 5, 12, 18, and 22 of the subscales intrinsic and integration, the factor controlled motivation included the four items of the subscale external, and the factor amotivation retained its original four items. In this publication, the analyses of both the first-order six- and three-factor model are presented. Our rate of missing data was 2.1% only. We removed five students from our analysis, according to list-wise deletion (31), who had missing data in at least one item of our behavioral regulation in cycling to and from school scale. Following this procedure, 234 students were eligible for analysis. In a confirmatory factor analysis, at least 200 participants are typically included to establish convergence and reliable results (32). As the method of estimation for our ordinal (33), non-normally distributed data (34) (first-order six-factor and second-order models: Mardia's coefficient = 135.424, critical ratio = 30.544; first-order three-factor model: Mardia's coefficient = 54.428, critical ratio = 22.711), we used unweighted least squares in line with previous research. In addition, measurement model invariance was tested (35) for our first-order six- and three-factor models under consideration of four steps (i.e., configural, metric/weak, scalar/strong, and residual/strict) (36) to control for measurement invariance across gender (eligible girls: *n* = 111, eligible boys: *n* = 121). As criteria for model fit, the goodness of fit (for good measurement properties: >0.95) and the standardized root mean square residual (for good measurement properties: <0.08) were set (37). The reason we decided to report two criteria for the model fit when performing a confirmatory factor analysis, although it is not required by the COnsensus-based Standards for the selection of health Measurement INstruments (37), is described in detail in the publication on the validation of the need satisfaction in cycling to and from school scale among students from Germany (15).

Convergent validity was assessed by calculating the average variance extracted in Excel (RRID: SCR_016137) (38), whereby values of at least 0.5 indicated good measurement properties (39). For this calculation, the standardized regression weights for each item of our first-order six- and three-factor models were used, which were obtained through the confirmatory factor analysis.

Discriminant validity was assessed by conducting a principal component analysis with Varimax, which is the most commonly used rotation method, using IBM SPSS Statistics (RRID: SCR_016479), V.29.0. This procedure revealed whether each item of our first-order six- and three-factor models loads on one factor only (32). In addition, we calculated and correlated the mean value for each factor of our first-order six- and three-factor models, using IBM SPSS Statistics (RRID: SCR_016479), V.29.0, to check the Fornell–Larcker criterion (also known as the AVE-SV approach). For good measurement properties, the square root of the factors' average variance extracted should be greater than the correlation between each factor of the tested models and any of the other five or two factors (39).

#### 2.3.2 Reliability

Internal consistency was calculated for all items of our first-order six- and three-factor models (intrinsic: *n* = 238, integration: *n* = 235, identification: *n* = 237, introjection: *n* = 236, external/controlled motivation: *n* = 237, amotivation: *n* = 236, autonomous motivation: *n* = 237) through Cronbach's alpha (29), by using IBM SPSS Statistics (RRID: SCR_016479), V.29.0. For each factor, Cronbach's alpha should be ≥0.7, and at least a low indication of enough structural validity was required for good measurement properties (37). In line with previous research, McDonald's Omega—an alternative method to estimate reliability—was not used for our ordinal data (40).

#### 2.3.3 Criterion validity

Concurrent criterion validity was calculated through multiple correlations between the mean value for each factor of our first-order six- and three-factor models and days per week of cycling to school usually (29), by using IBM SPSS Statistics (RRID: SCR_016479), V.29.0, to test our theoretical model suggested in previous research (17). For good measurement properties, the correlation coefficient should be ≥0.7 (37). In addition, binary logistic regressions were calculated to examine the associations between the mean value for each factor of our first-order six- and three-factor models as an independent variable (intrinsic: *n* = 232, integration: *n* = 229, identification: *n* = 231, introjection: *n* = 230, external/controlled motivation: *n* = 231, amotivation: *n* = 230, and autonomous motivation *n* = 231) and the occurrence of cycling to and from school (coded as “yes” vs. “no” based on responses to the item “usual mode of transportation to school” in our questionnaire) as a dependent variable. In all separate analyses for each factor of our tested models, the predicted probability referred to cycling to and from school coded as “yes.”

## 3 Results

The sociodemographic characteristics of the 239 participating students (girls: 46.9%, boys: 51.9%, diverse: 1.3%) aged 10–17 are described in detail in the publication on the validation of the need satisfaction in cycling to and from school scale (15) as both scales were provided to the same sample at the same time.

Students' mean perceived intrinsic motivation was 3.1 ± 1.3, perceived integrated motivation was 2.6 ± 1.2, perceived identified motivation was 2.8 ± 1.2, perceived introjected motivation was 1.4 ± 0.6, perceived external/controlled motivation was 1.7 ± 0.9, perceived amotivation was 2.1 ± 1.2 (15), and perceived autonomous motivation was 3.0 ± 1.3.

Mann–Whitney U tests revealed a gender difference in favor of boys compared with girls regarding mean intrinsic motivation (*p* = 0.008; boys: 3.3 ± 1.2, girls: 2.9 ± 1.2), mean integrated motivation (*p* = 0.003; boys: 2.9 ± 1.3, girls: 2.4 ± 1.1), and mean autonomous motivation (*p* = 0.003; boys: 3.2 ± 1.3, girls: 2.7 ± 1.2). No differences were found regarding mean identified motivation (*p* = 0.322), mean introjected motivation (*p* = 0.947), mean external/controlled motivation (*p* = 0.908), and mean amotivation (*p* = 0.156).

### 3.1 Construct validity

#### 3.1.1 First-order six-factor and second-order models

The first-order six-factor confirmatory factor analysis provided a goodness of fit of 0.993 and a standardized root mean square residual of 0.0556. However, standardized regression weights of the items “I feel ashamed when I do not cycle to and from school” (0.301), “I feel like a loser when I have not cycled to and from school” (0.400), and “I feel bad when I do not cycle to and from school” (0.544) within the factor introjection were small to moderate (see Supplementary Figure A.1 and Table A.2). The same applies to the item “it is important to make an effort to cycle to and from school” (0.615) within the factor identification and the item “I feel pressured by my friends and/or my family to cycle to and from school” (0.617) within the factor external. In Table 1, the results of the measurement model invariance tests across gender for the first-order six-factor model are presented, which show good measurement properties in all four models for the goodness of fit and, except for the model factor variance invariance (0.0839), good measurement properties for the standardized root mean square residual in all models.

**Table 1:** Measurement model invariance tests across gender for the first-order six-factor model.

Models	Goodness of fit	Standardized root mean square residual
Configural invariance	0.989	0.0632
Metric invariance	0.981	0.0724
Error variance invariance	0.971	0.0731
Factor variance invariance	0.973	0.0839

The average variance extracted was 0.774 for intrinsic motivation, 0.701 for integrated motivation, 0.582 for identified motivation, 0.283 for introjected motivation, 0.581 for external motivation, and 0.639 for amotivation.

The rotated component matrix of the principal component analysis showed a four-factor structure: (1) apart from one item, all items within the factors intrinsic, integration, and identification; (2) all items within the factor amotivation, whereby the item “I appreciate the benefits” within the factor identification loads on this subscale as well (0.584); (3) all items within the factor external; and (4) all items within the factor introjection. The correlations between the factors intrinsic and integration as well as integration and identification were larger than the square root of the average variance extracted of integration and identification, respectively (see Supplementary Table A.3). With regard to the remaining factors—intrinsic, introjection, external, and amotivation—their square root of the average variance extracted was greater than their correlations with any of the other five factors, respectively.

The second-order confirmatory factor analysis provided a goodness of fit of 0.989 and a standardized root mean square residual of 0.0630 (see Supplementary Figure A.2).

#### 3.1.2 First-order three-factor model

The first-order three-factor confirmatory factor analysis provided a goodness of fit of 0.997 and a standardized root mean square residual of 0.0313. However, standardized regression weights of the item “I feel pressured by my friends and/or my family to cycle to and from school” (0.649) within the factor controlled motivation was moderate (see Supplementary Figure A.3 and Table A.4). In Table 2, the results of the measurement model invariance tests across gender for the first-order three-factor model are presented, showing good measurement properties in all four models for the goodness of fit and the standardized root mean square residual.

**Table 2:** Measurement model invariance tests across gender for the first-order three-factor model.

Models	Goodness of fit	Standardized root mean square residual
Configural invariance	0.994	0.0426
Metric invariance	0.990	0.0531
Error variance invariance	0.986	0.0556
Factor variance invariance	0.987	0.0596

The average variance extracted was 0.762 for autonomous motivation, 0.582 for controlled motivation, and 0.639 for amotivation.

The rotated component matrix of the principal component analysis confirmed the three-factor structure. The correlations between the factors autonomous motivation, controlled motivation, and amotivation were not larger than their square root of the average variance extracted, respectively (see Supplementary Table A.5).

### 3.2 Reliability

A good interitem correlation with Cronbach's alpha ranging from 0.794 (identification) over 0.846 (external/controlled motivation), 0.875 (amotivation), 0.905 (integration), 0.927 (autonomous motivation) to 0.932 (intrinsic) was found. However, the four items within the factor introjection showed a too small interitem correlation with a Cronbach's alpha of 0.665, whereby this value did not increase independent of which item was removed. Furthermore, statistics revealed that Cronbach's alpha increased to 0.811 if the item “it is important to make an effort to cycle to and from school” within the factor identification was removed on the ground that its performance in the corrected item-total correlation was insufficient (0.544).

### 3.3 Criterion validity

The mean value for each factor of the first-order six- and three-factor models correlated significantly with days per week of cycling to school usually (*p* < 0.001), but the correlation coefficient was slightly under 0.7 (*R* = 0.641 and *R* = 0.616, respectively). Mean integrated motivation (*p* = 0.059) was not a unique predictor, but mean intrinsic motivation (*p* = 0.002), mean identified motivation (*p* = 0.026), mean introjected motivation (*p* = 0.008), mean external motivation (*p* = 0.027), mean amotivation (*p* < 0.001), mean autonomous motivation (*p* < 0.001), and mean controlled motivation (*p* < 0.001) were unique predictors. In addition, mean amotivation decreased the likelihood of cycling to school (*p* < 0.001; OR = 0.3 [CI 95 for OR: 0.2, 0.5]). The mean value for all other factors of the first-order six- and three-factor models increased the likelihood of cycling to school (for mean intrinsic motivation: *p* < 0.001; OR = 2.3 [CI 95 for OR: 1.8, 3.1], for mean integrated motivation: *p* < 0.001; OR = 2.1 [CI 95 for OR: 1.6, 2.8], for mean identified motivation: *p* < 0.001; OR = 2.9 [CI 95 for OR: 2.1, 4.1], for mean introjected motivation: *p* < 0.001; OR = 4.4 [CI 95 for OR: 2.5, 7.7], for mean external/controlled motivation: *p* < 0.001; OR = 1.7 [CI 95 for OR: 1.3, 2.4], and for mean autonomous motivation: *p* < 0.001; OR = 2.5 [CI 95 for OR: 1.9, 3.3]) (see Supplementary Table A.6).

## 4 Discussion

This study aimed at providing a scale that allows one to gain a deeper understanding of the motivational processes underlying cycling to and from school, by undertaking the following three steps: (a) translation of the original Spanish behavioral regulation in active commuting to and from school questionnaire into German, (b) adaptation of the questionnaire to the context of cycling to and from school, and (c) validation of the newly developed scale in terms of construct and criterion validity as well as reliability among students.

Students' perceived mean integrated, identified, introjected, and external/controlled motivation, as well as amotivation, were (somewhat) low, whereas an inconclusive tendency was found in mean intrinsic and autonomous motivation. The inconclusive finding regarding intrinsic motivation was consistent with one of our previous studies conducted in 2019/2020, which examined 136 students aged 12–15 from Germany (41).

### 4.1 Gender differences

In this study, it was found that less girls than boys cycled to school (26.6% vs. 39.7%) and girls cycled to school less frequently than boys (1.5 ± 2.1 vs. 2.0 ± 2.2 days per week), possibly because girls compared with boys perceived themselves as less competent (3.9 ± 1.1 vs. 4.2 ± 1.1) and autonomous (3.0 ± 1.0 vs. 3.4 ± 1.2) (15). Explanations for the gender differences in need satisfaction among students from Germany have been provided in our recent publication on the validation of the need satisfaction in cycling to and from school scale (15). The gender differences in this publication in favor of boys compared with girls regarding mean intrinsic, integrated, and autonomous motivation support our theoretical model suggested in previous research that need satisfaction leads to a more self-determined form of motivation, which, in turn, leads to (more) cycling to and from school (17).

### 4.2 Measurement properties

#### 4.2.1 First-order six-factor and second-order models

The first-order six-factor model of our behavioral regulation in cycling to and from school scale provided insufficient measurement properties for construct validity and reliability because of serious issues that forbid its application. The authors of the related scales on behavioral regulation in active travel to and from school from Spain (22), Portugal (23), and Sweden (24) reported better results.

Because of the good global model fit provided by the confirmatory factor analysis, sufficient structural validity was achieved for both the first-order six-factor model and the second-order model. However, standardized regression weights in the first-order six-factor model were small to moderate for three out of four items within the subscale introjection (i.e., items 2, 8, and 16), ranging from 0.301 over 0.400 to 0.544, and moderate for item 17 within the subscale identification (0.615) and item 19 within the subscale external (0.617), respectively. Even though no consensus has yet been reached on the proper value of the currently argued standardized regression weights (i.e., >0.4, 0.5 or 0.7) (32), local modal fit for at least two items within the subscale introjection with values ≤0.4 is doubtful. Although the standardized root mean square residual of 0.0839 for the model factor variance invariance in the first-order six-factor model was slightly above the critical value for good measurement properties of <0.08, as shown in the measurement model invariance tests, we still consider the factor structure of this model suitable for both genders. The minimal violation of the critical value appeared to be insignificant, as the goodness of fit, which is another criterion for model fit, was above the high cutoff point of 0.95 in all four models. Based on the average variance extracted, convergent validity was supported for the subscales, intrinsic, integrated, identified, and external motivation as well as amotivation, but not for introjected motivation in the first-order six-factor model. This indicates that the items within the subscale introjection did not measure its construct as intended. Based on the Fornell–Larcker criterion, discriminant validity was established for the subscales, intrinsic, introjection, external, and amotivation, but not for integration and identification in the first-order six-factor model, suggesting that these two subscales are related to/not distinct from others, and therewith, they are no stand-alone subscales. This assumption was further strengthened by the results of the principal component analysis of the first-order six-factor model that revealed a four-factor structure only, combining the subscales of intrinsic, integration, and identification in one factor. In this context, we also found a high cross-loading of item 3 within the subscale identification with amotivation, indicating that this item did not measure what it intended to measure. This finding suggests that the appreciation of the benefits of cycling to and from school is not enough to motivate students to engage in this specific behavior. While physical activity–related health benefits are well known (42), the prevalence of physical activity in children and adolescents from Germany remains low (13). Children and adolescents may be unconcerned about their future health, especially if they do not have any health problems as yet. Because of their stage of development, they may not be able to anticipate future consequences caused by their current behavior. Instead, they may just live for the moment. Despite the evidence of sufficient structural validity for the first-order six-factor model, reliability via internal consistency was not demonstrated because of a too small interitem correlation within the subscale introjection that could not be resolved. In addition, item 17 within the subscale identification should be removed because of its insufficient performance in the corrected item-total correlation. However, a subscale should not consist of only two items. Concurrent criterion validity is acceptable. Although the correlation between the mean value for each factor of the first-order six-factor model and days per week of cycling to school usually did not provide sufficiently good measurement properties (*R* = 0.641), it pointed in the expected direction. Furthermore, it needs to be noted that this analysis included a negative influence, like amotivation. The finding regarding the associations between the mean value for all factors of the first-order six-factor model and the occurrence of cycling to school, suggesting that intrinsic and extrinsic motivation (referring to the subscales external, introjected, identified, and integrated) are positive predictors of this specific behavior, is partly in line with previous research on active travel to school in Spain (22) and Sweden (24). In both validation studies, only intrinsic, integrated, and identified motivation were found to be positive predictors of active travel to school. Our finding highlights the need for internalizing the merit of cycling to and from school in this population as it suggests that students use their bike on the way to and from school not only because they like or value it or consider it important but also because they want to avoid negative feelings (e.g., shame) or feeling pressured by others (e.g., their friends, family, or teachers) (19, 20).

#### 4.2.2 First-order three-factor model

Overall, the first-order three-factor model of our behavioral regulation in cycling to and from school scale provided good measurement properties for construct and concurrent criterion validity and reliability without revealing any major limitations, which need to be considered before its application.

Because of the good global model fit provided by the confirmatory factor analysis, sufficient structural validity was achieved. The standardized regression weights of item 19 within the subscale controlled motivation was only moderate (i.e., 0.649); however, it was above two of the three critical values discussed in existing research (i.e., >0.4 and 0.5 but not 0.7) (32). Therefore, we do not expect this finding to have a negative impact on the validation of this scale. As shown in the measurement model invariance tests, the factor structure of this model was suitable for both genders. Based on the average variance extracted, convergent validity was supported. In addition, discriminant validity was established based on the Fornell–Larcker criterion and the results of the principal component analysis that confirmed the three-factor structure. Considering the evidence of sufficient structural validity, reliability via internal consistency of each subscale was proven. Concurrent criterion validity was acceptable. Although the correlation between the mean value for each factor of the first-order three-factor model and days per week of cycling to school usually did not provide sufficiently good measurement properties (*R* = 0.616), it pointed in the expected direction. Furthermore, it needs to be noted that this analysis included a negative influence of amotivation. The finding regarding the associations between the mean value for all factors of the first-order three-factor model and the occurrence of cycling to school, suggesting that autonomous (including items of the subscales intrinsic and integrated) and controlled motivation (including items of the subscale external) are positive predictors of this specific behavior, was observed inconsistently in existing research in terms of controlled motivation (22, 24, 43). In the Spanish and Swedish validation study, intrinsic and integrated motivation were positive predictors of active travel to school; however, external motivation was a negative but insignificant predictor in Spain and a positive but insignificant predictor in Sweden (22, 24). A Chinese study found that both internal and external motivation played a role in cycling behavior, whereby external motivation appeared to be less important (43). Our finding emphasizes the need for internalizing the value of cycling to and from school in our target group as it suggests that students use their bike for going to and returning from school not only because they enjoy it or consider it an important part of themselves but also because they strive for meeting social demands (e.g., demands from their friends, family, or teachers) (19, 20).

### 4.3 Practical implications

The use of a validated scale assessing the motivation of cycling to and from school supports the understanding of how students can be encouraged in engaging in this specific behavior (more regularly) so that practitioners, such as (physical education) teachers, or researchers are able to implement effective and evaluate tailored interventions that respond to the students' actual needs and contexts. Moreover, (physical education) teachers can also benefit from the information provided by this scale to group students when providing specific tasks/activities that align with the students' motivational profiles. In terms of the students' motivational profiles, this scale allows researchers to gain a deeper understanding of their diversity and how they are influenced by other critical variables such as sociodemographic characteristics, parental attitudes and behaviors, and perceptions of safety, time, and so on.

### 4.4 Limitations

The limitations of this study are described in detail in the publication on the validation of the need satisfaction in cycling to and from school scale among students from Germany (15). In summary, weaknesses refer to a self-selection bias of schools limiting the generalization of our findings, unclear impacts of Covid-19 (e.g., homeschooling) and seasons (because of two data collection waves as a result of recruitment challenges), a lack of gender-inclusive research in terms of diversity due to an insufficient sample size, the risk of a social-desirability bias resulting from the type of our data collection method, choosing a cross-sectional design considering practicality (i.e., an analysis of test–retest reliability rendered unfeasible), and not using multiple translation designs (i.e., an additional backward translation) for reasons of expedience.

## 5 Conclusions

This study provides the first valid and reliable scale developed to measure behavioral regulation specifically in cycling to and from school among children and adolescents in Germany, by applying a first-order three-factor model.

As we found a central tendency bias in intrinsic and autonomous motivation, we suggest re-evaluating the use of a five-point Likert scale. Future research is also needed to: (a) develop a scale measuring behavior regulation in cycling to and from school, by using a first-order six-factor model, (b) investigate the influence of extrinsic and controlled motivation on cycling to and from school in the long term, (c) test the effectiveness of already suggested interventions, which may establish a more self-determined motivation through need satisfaction particularly in girls (15) but also in both genders (17) as a promising way to encourage them to engage in cycling to and from school (more often), and (d) demonstrate cross-cultural validity of our scale (e.g., for a cross-country comparison of motivational profiles).

## Data Availability

The raw data supporting the conclusions of this article will be made available by the authors without undue reservation.

## References

[1] United Nations. 68% of the world population projected to live in urban areas by 2050, says UN. (2018). Available online at: https://www.un.org/development/desa/en/news/population/2018-revision-of-world-urbanization-prospects.html (Accessed September 1, 2025).

[2] United Nations. Goal 11—make cities and human settlements inclusive, safe, resilient and sustainable. (n.d.). Available online at: https://sdgs.un.org/goals/goal11 (Accessed September 1, 2025).

[3] United Nations. Sustainable transport. (n.d.). Available online at: https://sdgs.un.org/topics/sustainable-transport (Accessed September 1, 2025).

[4] World Health Organization. Physical activity strategy for the WHO European Region 2016–2025. (2016). Available online at: https://iris.who.int/bitstream/handle/10665/329407/9789289051477-eng.pdf?sequence=1 (Accessed September 1, 2025).

[5] United Nations. Goal 3—ensure healthy lives and promote well-being for all at all ages. (n.d.). Available online at: https://sdgs.un.org/goals/goal3 (Accessed September 3, 2025).

[6] United Nations. Goal 13—take urgent action to combat climate change and its impacts. (n.d.). Available online at: https://sdgs.un.org/goals/goal13 (Accessed September 3, 2025).

[7] World Health Organization. Unlocking new opportunities. Jobs in green and healthy transport. (2014). Available online at: https://iris.who.int/handle/10665/344592 (Accessed September 1, 2025).

[8] Logan G Somers C Baker G Connell H Gray S Kelly P Benefits, risks, barriers, and facilitators to cycling: a narrative review. Front Sports Act Living. (2023) 5:1168357. 10.3389/fspor.2023.1168357 37795314 PMC10546027

[9] Federal Ministry for Digital and Transport. Germany 2023—a cycling nation. National cycling plan 3.0. (2022). Available online at: https://www.bmv.de/SharedDocs/DE/Anlage/StV/nationaler-radverkehrsplan-3-0-en.pdf?__blob=publicationFile#:∼:text=Cycling%20is%20healthy%2C%20cheap%20and,the%20fastest%20means%20of%20transport (Accessed January 3, 2026).

[10] Vanpée R Van Zeebroeck B. A comparative cost-benefit analysis of cycling within the Benelux and North Rhine-Westphalia. (2022). Available online at: https://www.benelux.int/wp-content/uploads/2023/03/Report_Cycling_Benelux_NRW.pdf (Accessed January 3, 2026).

[11] Roth MA Millett CJ Mindell JS. The contribution of active travel (walking and cycling) in children to overall physical activity levels: a national cross sectional study. Prev Med. (2012) 54:134–9. 10.1016/j.ypmed.2011.12.004 22182478

[12] Aubert S Barnes JD Demchenko I Hawthorne M Abdeta C Abi Nader P Global matrix 4.0 physical activity report card grades for children and adolescents: results and analyses from 57 countries. J Phys Act Health. (2022) 19:700–28. 10.1123/jpah.2022-0456 36280233

[13] Demetriou Y Beck F Sturm D Abu-Omar K Forberger S Hebestreit A Germany’s 2022 report card on physical activity for children and adolescents. Ger J Exerc Sport Res. (2024) 54:260–75. 10.1007/s12662-024-00946-6

[14] Bundesministerium für Verkehr und digitale Infrastruktur. Radverkehr in Deutschland. Zahlen, Daten, Fakten. (2014). Available online at: https://www.bmv.de/SharedDocs/DE/Publikationen/K/radverkehr-in-zahlen.pdf?__blob=publicationFile (Accessed January 3, 2026).

[15] Schönbach DMI Marques A Peralta M Kleszczewska D Dzielska A Burgueño R Validation of the need satisfaction in cycling to and from school scale among students from Germany. J Cycl Micromobility Res. (2025) 3:100052. 10.1016/j.jcmr.2024.100052

[16] Schönbach DMI Vondung C Hidding LM Altenburg TM Chinapaw MJM Demetriou Y. Gender influence on students, parents, and teachers’ perceptions of what children and adolescents in Germany need to cycle to school: a concept mapping study. Int J Environ Res Public Health. (2020) 17:6872. 10.3390/ijerph17186872 32962261 PMC7557880

[17] Schönbach DMI Chillón P Marques A Peralta M Demetriou Y. Study protocol of a school-based randomized controlled trial to promote cycling to school among students in Germany using intervention mapping: the ACTS project. Front Public Health. (2021) 9:661119. 10.3389/fpubh.2021.661119 34434911 PMC8380952

[18] Ryan RM Deci EL. Self-Determination Theory. Basic Psychological Needs in Motivation, Development and Wellness. New York: The Guilford Press (2017). p. 756.

[19] Ryan RM Deci EL. Intrinsic and extrinsic motivation from a self-determination theory perspective: definitions, theory, practices, and future directions. Contemp Educ Psychol. (2020) 61:101860. 10.1016/j.cedpsych.2020.101860

[20] Ryan RM Deci EL Vansteenkiste M Soenens B. Building a science of motivated persons: self-determination theory’s empirical approach to human experience and the regulation of behavior. Motiv Sci. (2021) 7:97–110. 10.1037/mot0000194

[21] Schönbach DMI Altenburg TM Marques A Chinapaw MJM Demetriou Y. Strategies and effects of school-based interventions to promote active school transportation by bicycle among children and adolescents: a systematic review. Int J Behav Nutr Phys Act. (2020) 17:138. 10.1186/s12966-020-01035-1 33183331 PMC7661215

[22] Burgueño R González-Cutre D Sevil-Serrano J Herrador-Colmenero M Segura-Díaz JM Medina-Casaubón J Understanding the motivational processes involved in adolescents’ active commuting behaviour: development and validation of the behavioural regulation in active commuting to and from school (BR-ACS) questionnaire. Transp Res Part F Traffic Psychol Behav. (2019) 62:615–25. 10.1016/j.trf.2019.02.016

[23] Marques A Santos T Demetriou Y Schönbach DMI Peralta M Lagestad P Adaptation of the behavioural regulation in active commuting to school (BR-ACS) questionnaire in Portuguese youth. Children. (2022) 9:182. 10.3390/children9020182 35204902 PMC8870428

[24] Burgueño R Rutberg S Nyberg L Pauelsen M Chillón P Lindqvist A-K. Adapting the behavioral regulation in active commuting to and from school questionnaire in Sweden: BR-ACS(SWE). Transp Res Interdiscip Perspect. (2022) 16:100721. 10.1016/j.trip.2022.100721

[25] International Test Commission. The ITC guidelines for translating and adapting tests (second edition). (2017). Available online at: www.InTestCom.org (Accessed September 1, 2025).

[26] Deutsche Forschungsgemeinschaft. FAQ: Humanities and social sciences. (2023). Available online at: https://www.dfg.de/en/research-funding/proposal-funding-process/faq/humanities-social-sciences (Accessed January 3, 2026).

[27] Technical University of Munich. Ethics committee. (n.d.). Available online at: https://www.tum.de/en/about-tum/organization/ethics-committee (Accessed January 3, 2026).

[28] COSMIN. Definitions of measurement properties – Order of importance. (n.d.). Available online at: https://www.cosmin.nl/tools/cosmin-taxonomy-measurement-properties/ (Accessed September 1, 2025).

[29] Mokkink LB Prinsen CAC Patrick DL Alonso J Bouter LM de Vet HCW COSMIN study design checklist for patient-reported outcome measurement instruments. (2019). Available online at: https://www.cosmin.nl/wp-content/uploads/COSMIN-study-designing-checklist_final.pdf (Accessed September 1, 2025).

[30] Owen KB Smith J Lubans DR Ng JYY Lonsdale C. Self-determined motivation and physical activity in children and adolescents: a systematic review and meta-analysis. Prev Med. (2014) 67:270–9. 10.1016/j.ypmed.2014.07.033 25073077

[31] Schober P Vetter TR. Missing data and imputation methods. Anesth Analg. (2020) 131:1419–20. 10.1213/ANE.0000000000005068 33079865 PMC7553195

[32] Cheung GW Cooper-Thomas HD Lau RS Wang LC. Reporting reliability, convergent and discriminant validity with structural equation modeling: a review and best-practice recommendations. Asia Pac J Manag. (2024) 41:745–83. 10.1007/s10490-023-09871-y

[33] Koğar H Koğar EY. Comparison of different estimation methods for categorical and ordinal data in confirmatory factor analysis. J Meas Eval Educ Psy. (2015) 6:351–64. 10.21031/epod.94857

[34] Zulkifli NR Aimran N Deni SM. The performance of unweighted least squares and regularized unweighted least squares in estimating factor loadings in structural equation modeling. Int J Data Netw Sci. (2023) 7:1017–24. 10.5267/j.ijdns.2023.6.004

[35] Milfont TL Fischer R. Testing measurement invariance across groups: applications in cross-cultural research. Int J Psychol Res. (2010) 3:111–21. 10.21500/20112084.857

[36] Putnick DL Bornstein MH. Measurement invariance conventions and reporting: the state of the art and future directions for psychological research. Dev Rev. (2016) 41:71–90. 10.1016/j.dr.2016.06.004 27942093 PMC5145197

[37] Mokkink LB Prinsen CAC Patrick DL Alonso J Bouter LM de Vet HCW COSMIN methodology for systematic reviews of patient-reported outcome measures (PROMs). User manual. (2018). Available online at: https://www.cosmin.nl/wp-content/uploads/COSMIN-syst-review-for-PROMs-manual_version-1_feb-2018.pdf (Accessed September 1, 2025). 10.1007/s11136-018-1798-3 PMC5891568 29435801

[38] Hair JF Black WC Babin BJ Anderson RE. Multivariate Data Analysis. London: Pearson (2013). p. 740.

[39] Fornell C Larcker DF. Evaluating structural equation models with unobservable variables and measurement error. J Mark Res. (1981) 18:39–50. 10.2307/3151312

[40] Orcan F. Comparison of Cronbach’s alpha and McDonald’s omega for ordinal data: are they different? Int J Assess Tool Educ. (2023) 10:709–22. 10.21449/ijate.1271693

[41] Hidding LM Chinapaw MJM Brindley C Demetriou Y Dzielska A Hamrik Z Perspectives from adolescents, parents, and teachers on barriers and facilitators for European adolescents to cycle to and from school: a concept mapping study. J Phys Act Health. (2025) 22:1000–12. 10.1123/jpah.2024-0508 40609982

[42] Poitras VJ Gray CE Borghese MM Carson V Chaput J-P Janssen I Systematic review of the relationships between objectively measured physical activity and health indicators in school-aged children and youth. Appl Physiol Nutr Metab. (2016) 41:197–239. 10.1139/apnm-2015-0663 27306431

[43] Xu H Yuan M Li J. Exploring the relationship between cycling motivation, leisure benefits and well-being. Int Rev Spat Plan Sustain Dev. (2019) 7:157–71. 10.14246/irspsda.7.2_157

