# Supplementary material for: Validation of the behavioral regulation in cycling to and from school scale among students from Germany
Source: Front Sports Act Living. 2026 Feb 11;8:1701435. doi: 10.3389/fspor.2026.1701435 (PMC12931929; doi:10.3389/fspor.2026.1701435)
Supplement: Supplementary file 1 [file Datasheet1.docx]

Table A.1: German behavioral regulation in cycling to and from school scale and its English translation.

| **Language** | | | **German** | **English** |
| --- | --- | --- | --- | --- |
| **Question** | | | Ich fahre oder würde normalerweise mit dem Fahrrad zur und von der Schule fahren, weil… | I normally cycle or would cycle to and from school because… |
| **Five-point Likert scale** | | | 1 (stimme überhaupt nicht zu), 2 (stimme eher nicht zu), 3 (teils, teils), 4 (stimme eher zu), 5 (stimme voll und ganz zu) | 1 (do not agree at all), 2 (rather do not agree), 3 (partly (do not) agree), 4 (rather agree), 5 (completely agree) |
| **Behavioral regulations** | **Intrinsic motivation** | 4 | es Spaß macht. | it is fun. |
|  |  | 12 | ich es genieße. | I enjoy it. |
|  |  | 18 | ich es als eine angenehme Aktivität empfinde. | I find it a pleasant activity. |
|  |  | 22 | ich es mag. | I like it. |
|  | **Integrated motivation** | 5 | es meinem Typ entspricht. | it is consistent with my type. |
|  |  | 10 | es ein Teil meiner Persönlichkeit ist. | it is part of my personality. |
|  |  | 15 | es ein wichtiger Teil von mir ist. | it is an important part of me. |
|  |  | 20 | es mit meinen eigenen Werten übereinstimmt. | it is in line with my own values. |
|  | **Identified motivation** | 3 | ich die Vorteile schätze. | I appreciate the benefits. |
|  |  | 9 | es mir wichtig ist. | it is important to me. |
|  |  | 17 | es wichtig ist, sich zu bemühen, mit dem Fahrrad zur und von der Schule zu fahren. | it is important to make an effort to cycle to and from school. |
|  | **Introjected motivation** | 2 | ich mich schlecht fühle, wenn ich nicht mit dem Fahrrad zur und von der Schule fahre. | I feel bad when I do not cycle to and from school. |
|  |  | 8 | ich mich schäme, wenn ich nicht mit dem Fahrrad zur und von der Schule fahre. | I feel ashamed when I do not cycle to and from school. |
|  |  | 16 | ich mich wie eine Loserin/ein Loser fühle, wenn ich nicht mit dem Fahrrad zur und von der Schule gefahren bin. | I feel like a loser when I have not cycled to and from school. |
|  |  | 21 | ich unruhig werde, wenn ich nicht mit dem Fahrrad zur und von der Schule fahre. | I get restless when I do not cycle to and from school. |
|  | **External motivation** | 1 | andere Leute sagen, dass ich mit dem Fahrrad zur und von der Schule fahren soll. | other people say that I should cycle to and from school. |
|  |  | 7 | meine Freundinnen/Freunde, meine Familie und/oder meine Lehrerin/mein Lehrer sagen/sagt, dass ich mit dem Fahrrad zur und von der Schule fahren soll. | my friends, my family and/or my teacher say(s) that I should cycle to and from school. |
|  |  | 13 | andere Leute mit mir unzufrieden sein werden, wenn ich nicht mit dem Fahrrad zur und von der Schule fahre. | other people will be displeased with me when I do not cycle to and from school. |
|  |  | 19 | ich mich von meinen Freundinnen/Freunden und/oder meiner Familie dazu gedrängt fühle, mit dem Fahrrad zur und von der Schule zu fahren. | I feel pressured by my friends and/or my family to cycle to and from school. |
|  | **Amotivation** | 6 | Ich verstehe nicht, warum ich mit dem Fahrrad zur und von der Schule fahren sollte. | I do not understand why I should cycle to and from school. |
|  |  | 11 | Ich verstehe nicht, warum ich mir die Mühe machen sollte, mit dem Fahrrad zur und von der Schule zu fahren. | I do not understand why I should make an effort to cycle to and from school. |
|  |  | 14 | Ich sehe keinen Sinn darin, mit dem Fahrrad zur und von der Schule zu fahren. | I do not see the point in cycling to and from school. |
|  |  | 23 | Ich denke, dass das Fahrradfahren zur und von der Schule Zeitverschwendung ist. | I think that cycling to and from school is a waste of time. |

Table A.2: Standardized regression weights for each item of the first-order six-factor model based on confirmatory factors analysis (*n* = 234).

| **Behavioral regulations** | **Item** | **Standardized regression weights** |
| --- | --- | --- |
| Intrinsic motivation | it is fun. | .869 |
|  | I enjoy it. | .886 |
|  | I find it a pleasant activity. | .873 |
|  | I like it. | .892 |
| Integrated motivation | it is consistent with my type. | .881 |
|  | it is part of my personality. | .775 |
|  | it is an important part of me. | .840 |
|  | it is in line with my own values. | .850 |
| Identified motivation | I appreciate the benefits. | .835 |
|  | it is important to me. | .819 |
|  | it is important to make an effort to cycle to and from school. | .615 |
| Introjected motivation | I feel bad when I do not cycle to and from school. | .544 |
|  | I feel ashamed when I do not cycle to and from school. | .301 |
|  | I feel like a loser when I have not cycled to and from school. | .400 |
|  | I get restless when I do not cycle to and from school. | .765 |
| External motivation | other people say that I should cycle to and from school. | .858 |
|  | my friends, my family and/or my teacher say(s) that I should cycle to and from school. | .815 |
|  | other people will be displeased with me when I do not cycle to and from school. | .736 |
|  | I feel pressured by my friends and/or my family to cycle to and from school. | .617 |
| Amotivation | I do not understand why I should cycle to and from school. | .766 |
|  | I do not understand why I should make an effort to cycle to and from school. | .844 |
|  | I do not see the point in cycling to and from school. | .836 |
|  | I think that cycling to and from school is a waste of time. | .746 |

Table A.3: Correlations between the factors of the first-order six-factor model (*n* = 234).

| **Behavioral regulations** | Mean intrinsic motivation | Mean integrated motivation | Mean identified motivation | Mean introjected motivation | Mean external motivation | Mean amotivation |
| --- | --- | --- | --- | --- | --- | --- |
| Mean intrinsic motivation | 1 |  |  |  |  |  |
| Mean integrated motivation | .844 (*p* < .001) | 1 |  |  |  |  |
| Mean identified motivation | .727 (*p* < .001) | .786 (*p* < .001) | 1 |  |  |  |
| Mean introjected motivation | .286 (*p* < .001) | .421 (*p* < .001) | .441 (*p* < .001) | 1 |  |  |
| Mean external motivation | -.008 (*p* = .905) | .043 (*p* = 508) | .136 (*p* = .037) | .320 (*p* < .001) | 1 |  |
| Mean amotivation | -.631 (*p* < .001) | -.570 (*p* < .001) | -.664 (*p* < .001) | -.212 (*p* = .001) | -.049 (*p* = .456) | 1 |

*p* = probability value

Table A.4: Standardized regression weights for each item of the first-order three-factor model based on confirmatory factors analysis (*n* = 234).

| **Factors** | **Item** | **Standardized regression weights** |
| --- | --- | --- |
| Autonomous motivation | it is consistent with my type. | .847 |
|  | I enjoy it. | .872 |
|  | I find it a pleasant activity. | .858 |
|  | I like it. | .914 |
| Controlled motivation | other people say that I should cycle to and from school. | .846 |
|  | my friends, my family and/or my teacher say(s) that I should cycle to and from school. | .807 |
|  | other people will be displeased with me when I do not cycle to and from school. | .736 |
|  | I feel pressured by my friends and/or my family to cycle to and from school. | .649 |
| Amotivation | I do not understand why I should cycle to and from school. | .777 |
|  | I do not understand why I should make an effort to cycle to and from school. | .851 |
|  | I do not see the point in cycling to and from school. | .820 |
|  | I think that cycling to and from school is a waste of time. | .745 |

Table A.5: Correlations between the factors of the first-order three-factor model (*n* = 234).

| **Factors** | Mean autonomous motivation | Mean controlled motivation | Mean amotivation |
| --- | --- | --- | --- |
| Mean autonomous motivation | 1 |  |  |
| Mean controlled motivation | .012 (*p* = .857) | 1 |  |
| Mean amotivation | -.626 (*p* < .001) | -.049 (*p* = .456) | 1 |

*p* = probability value

Table A.6: Binary logistic regressions of the mean value for each factor of the two tested models and occurrence of cycling to and from school.

| **Behavioral regulations/factors** | ***p*** | **OR** | **95% CI for OR** | |
| --- | --- | --- | --- | --- |
|  |  |  | **Lower** | **Upper** |
| Mean intrinsic motivation | < .001 | 2.3 | 1.8 | 3.1 |
| Mean integrated motivation | < .001 | 2.1 | 1.6 | 2.8 |
| Mean identified motivation | < .001 | 2.9 | 2.1 | 4.1 |
| Mean introjected motivation | < .001 | 4.4 | 2.5 | 7.7 |
| Mean external/controlled motivation | < .001 | 1.7 | 1.3 | 2.4 |
| Mean amotivation | < .001 | .3 | .2 | .5 |
| Mean autonomous motivation | < .001 | 2.5 | 1.9 | 3.3 |

CI = confidence interval; OR = odds ratio; *p* = probability value


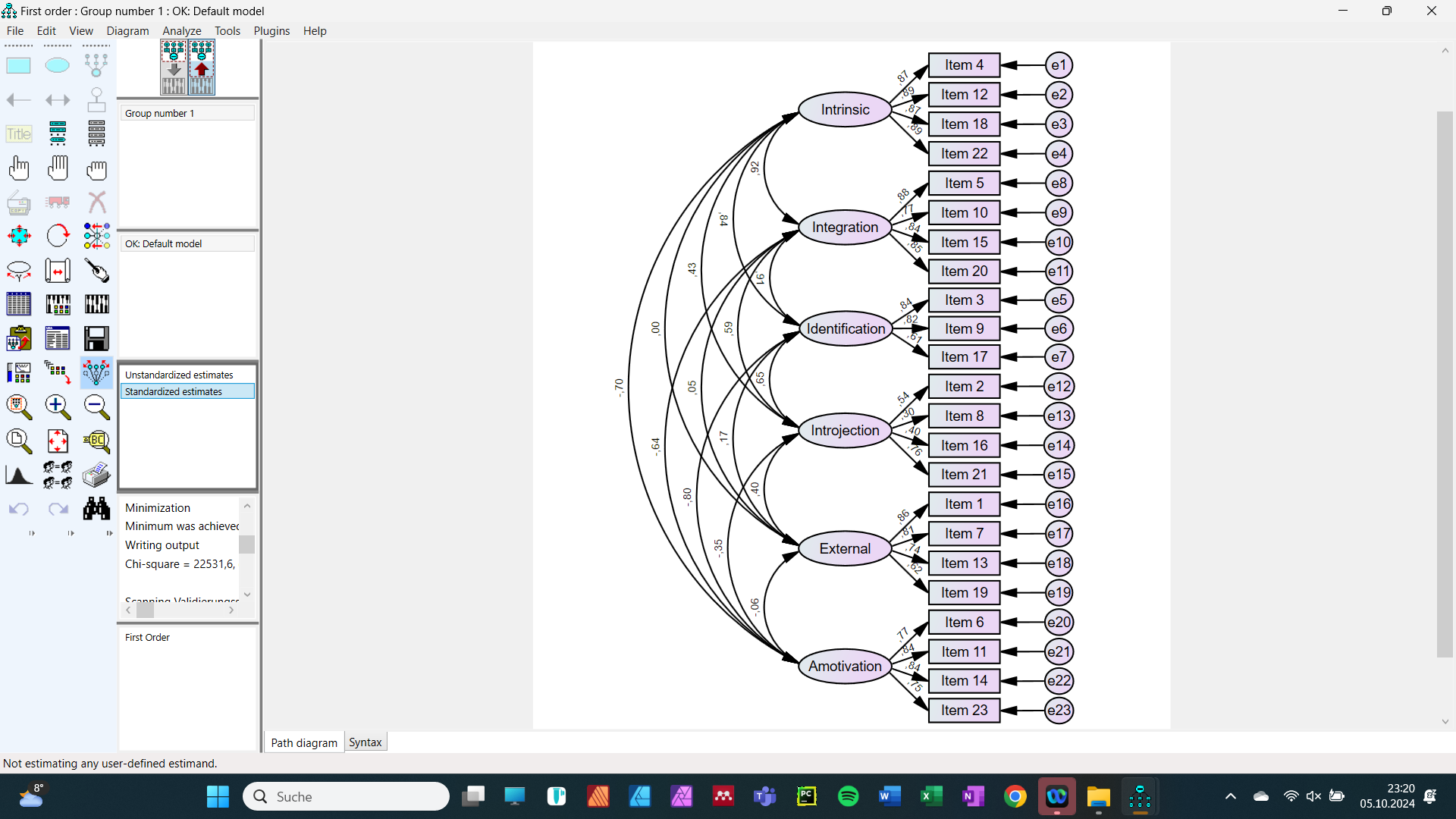


Figure A.1: First-order six-factor confirmatory factor analysis of the behavioral regulation in cycling to and from school scale (*n* = 234).


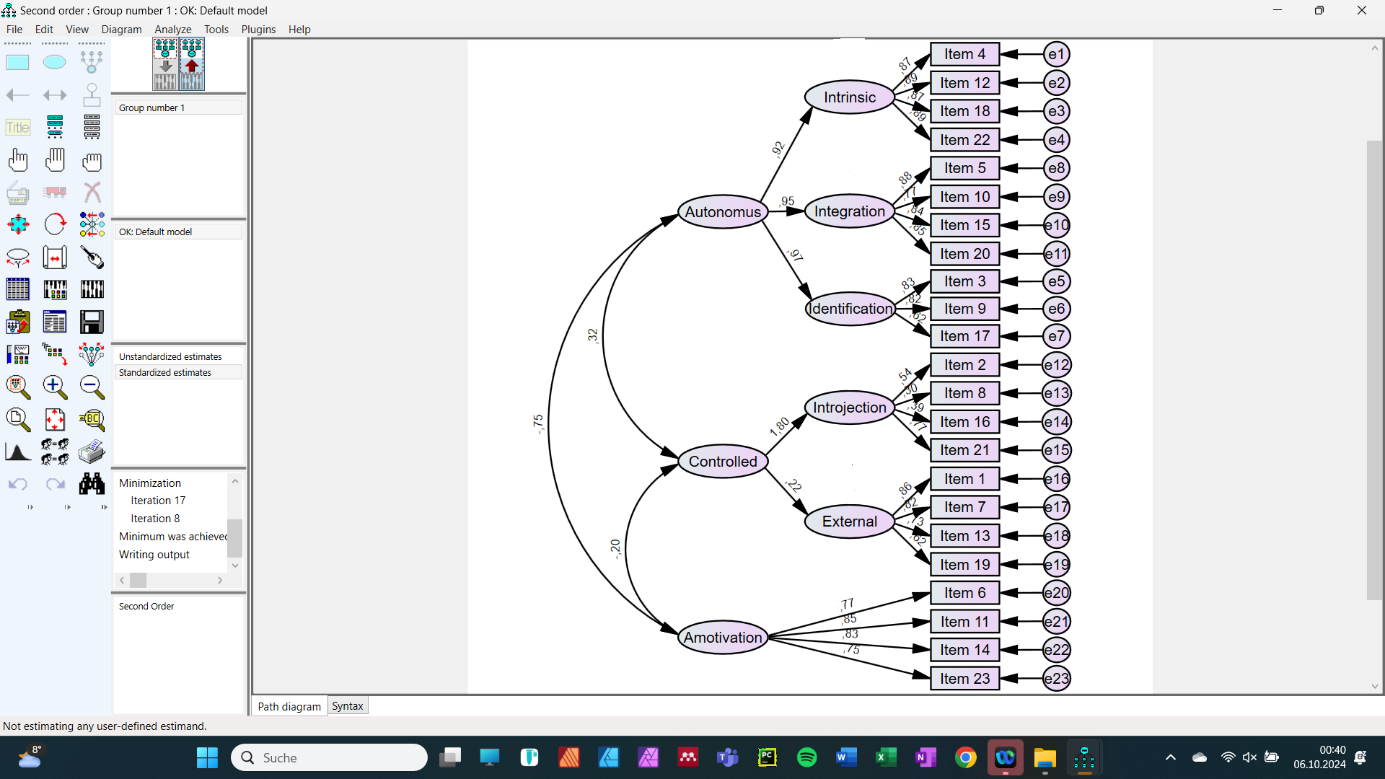


Figure A.2: Second-order confirmatory factor analysis of the behavioral regulation in cycling to and from school scale (*n* = 234).


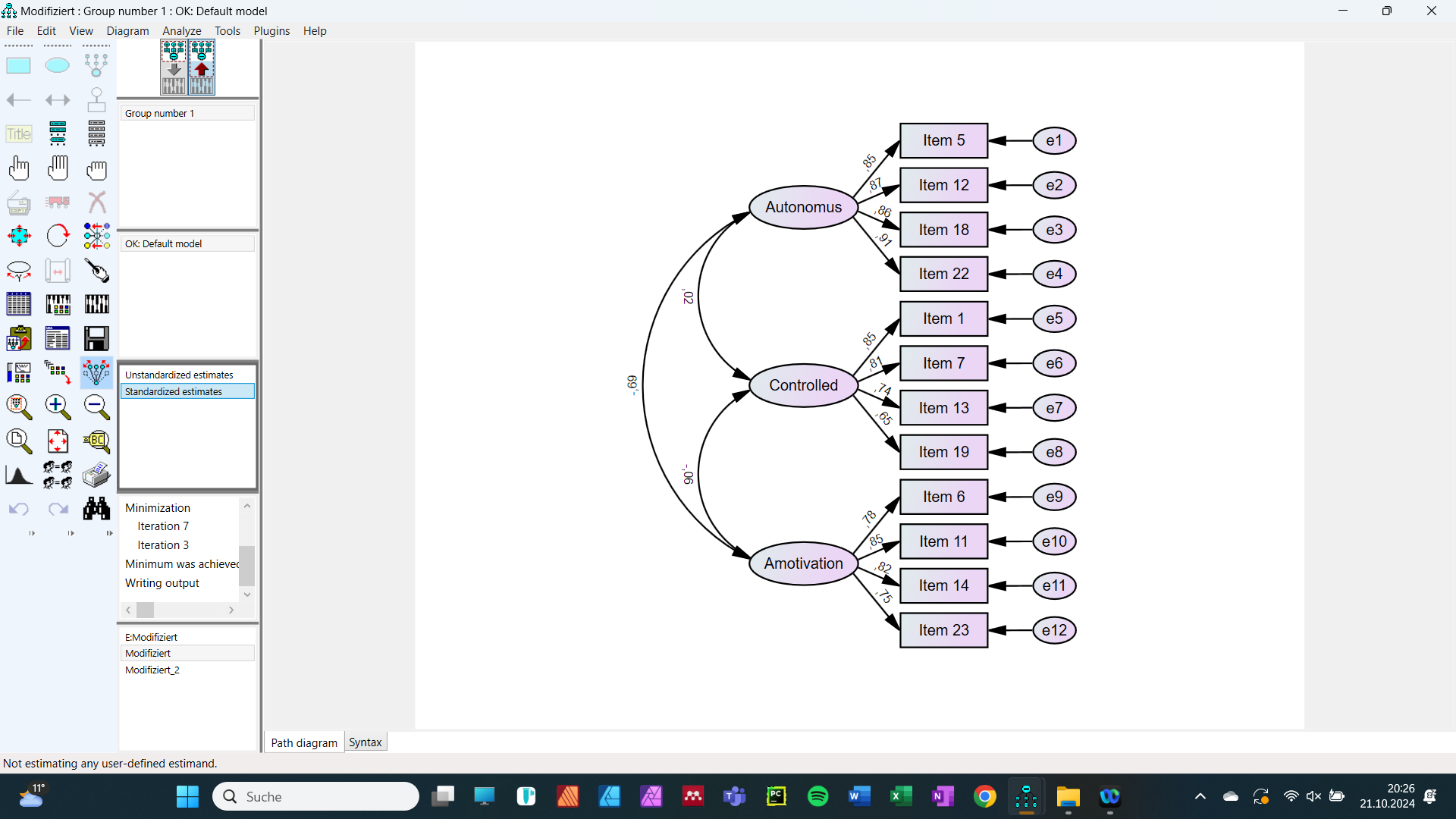


Figure A.3: First-order three-factor confirmatory factor analysis of the behavioral regulation in cycling to and from school scale (*n* = 234).
